# Prevalence and Associated Factors of Depressive Symptoms Among Adults During Armed Conflict in Sudan: The Role of Internal Displacement

**DOI:** 10.3390/healthcare14152248

**Published:** 2026-07-23

**Authors:** Ferdows K. Kheiri, Sufian K. Noor, Sami S. Alharthi, Hatim Y. Alharbi, Afnan A. Alwabili, Ishag Adam

**Affiliations:** 1 Department of Medicine, Faculty of Medicine, Nile Valley University, Atbara 46611, Sudan; ferdowskheiri98@gmail.com (F.K.K.); sufiankhalid@yahoo.com (S.K.N.); 2 Department of Medicine, College of Medicine, Taif University, Taif 21944, Saudi Arabia; s.s.alharthi@tu.edu.sa; 3 Department of Psychiatry, College of Medicine, Qassim University, Buraydah 52571, Saudi Arabia; hy.alharbi@qu.edu.sa; 4 Department of Obstetrics and Gynecology, College of Medicine, Qassim University, Buraydah 52571, Saudi Arabia

**Keywords:** internal displacement, depression, age, adults, female

## Abstract

**Background:** Mental health disorders, including depression, constitute a major global public health concern that can lead to significant morbidity as well as societal costs. Internally displaced people are at risk for depression. This study aimed to assess the prevalence of depressive symptoms among adults in Atbara, northern Sudan, and identify the key factors associated with depressive symptoms, including internal displacement. **Methods:** Using a stratified sampling technique, data were collected by trained research assistants through face-to-face interviews using a structured questionnaire. The Patient Health Questionnaire-9 (PHQ-9) was used to assess depressive symptoms. Multivariate binary analysis was performed. **Results:** Three hundred and seventy-three adults were enrolled in this study; 104 (27.9%) were males. Their median age was 42.0 years. Of the 373 adults, 81 (21.7%, 95.0% confidence interval = 17.5–25.9%) had depressive symptoms (PHQ-9 scores ≥ 8). The prevalence of depressive symptoms was significantly higher among displaced adults: 39 (28.1%) of the 139 displaced adults had depression, and 42 (17.9%) of the non-displaced adults had depression, *p* = 0.027. In multivariate binary analysis, being internally displaced was the only factor associated with depressive symptoms (adjusted odds ratio = 1.82, 95% confidence interval = 1.07–3.08); age, body mass index, sex, education, and marital status were not associated with depressive symptoms. **Conclusions:** One out of five adults in this region of Sudan had depressive symptoms, which was associated with internal displacement. More effort and further research are needed to explore possible interventions to improve mental health and livelihoods, especially among internally displaced people.

## 1. Introduction

Mental health illness, including depression and anxiety, is one of the major global public health concerns that can lead to significant morbidity as well as societal costs [1,2,3,4]. It has been estimated that globally, 5% and 4% of adults suffer from depression and anxiety, respectively [2,3]. While these conditions are prevalent worldwide, they are more likely to occur, and their impact is particularly severe, in populations and regions such as Sub-Saharan Africa (SSA), which are affected by wartime conflicts and forced displacement [4,5]. The war trauma, home instability, and socioeconomic hardships associated with war effects create ground for the development and exacerbation of mental health disorders, even after displaced persons return to their hometowns (i.e., returnee displaced populations). Mental health illnesses, such as depression and anxiety, have several short-term and long-term adverse effects and impacts, including poor quality of life and increased rates of suicide [3,4]. Fortunately, effective treatments are available for these mental health disorders, especially if they are discovered and effectively managed [2,3]. 

The prevalence of depression among populations varies in different settings in Africa, including Sudan [1,6,7]. For example, in a large community-based study, 25.2% of 2456 adults in Ghana had depression [8]. Likewise, 17.5% of the adults in Ethiopia had depression [6]. Several factors, such as age [6,9], being female [1,2,6,9,10], unmarried status [6,9], high body mass index (BMI) [11,12,13], having a chronic disease such as diabetes mellitus [14] or hypertension [2,3], having a low education level [8,10], unemployment [9,10], smoking habit [6], and alcohol consumption [2,15], have been reported to be associated with depression in adults in different populations. 

In addition to the above-mentioned traditional factors for depression, several recent studies in different populations have shown that displaced people are at risk for depression [16,17,18,19,20,21,22,23]. For example, in Ukraine, 13.9% of adults had depression before the war (2022), while 37.0% of them had moderate-to-severe symptoms of depression after the war and displacement [21]. Likewise, in 2023, 63.8% of armed conflict survivors in the northern region of Mozambique had depression [19]. In England, people who were displaced by flooding were at higher risk for depression than those flooded, but not displaced [17]. 

Sudan is the third largest African country and is currently facing an ongoing war, presenting a particularly critical context for mental health research. The war has led to widespread displacement of the population, as well as loss of livelihoods, psychological distress, and increased rates of mental health issues [10]. It has been observed that the prevalence of post-traumatic stress disorder (PTSD) was more common among displaced compared with non-displaced individuals in Sudan [9]. The loss of relatives, destruction of wealth, and displacement circumstances have exposed Sudanese populations to several mental health disorders [10]. However, despite the significant burden imposed by war, data on the mental health status of the Sudanese population and mental health services remain scarce, and in fact, Sudan was not prepared to deal with the extraordinary situations created by combat. Actually, Sudan faced several challenges and shortcomings in mental health even before the war [24,25], and there are unmet mental health needs [24,26]. Some previous studies that were conducted during wartime were conducted on the displaced population in camps, and some of them were online surveys [9,10,27,28,29]. Little is known regarding depression in the community, including both internally displaced and non-internally displaced. Thus, the present study was conducted to assess the prevalence of depressive symptoms among adults in Atbara, northern Sudan, and identify the key factors associated with the depressive symptoms, including internal displacement.

## 2. Materials and Methods

### 2.1. Study Design and Setting

Atbara is a major industrial center in River Nile State, northern Sudan, and the capital of Atbara locality. It has been estimated that, with a pre-conflict population of 110,000, Atbara hosts over 144,000 internally displaced persons (IDPs), which has more than doubled since April 2023. Internally displaced people primarily originate from the capital, Khartoum, Khartoum North, Gezira, and Sennar. Thus, services, including health delivery, have come under extreme pressure due to the ongoing national conflict [30]. The Strengthening the Reporting of Observational Studies in Epidemiology (STROBE) guidelines were followed in this study [31].

### 2.2. Study Population and Sampling

Using a stratified multistage sampling technique, five of the thirteen districts (Al-Hasaya, Hay-Alsharqi, Alfaki Madani, Al-Dakhla, and the Railway District) were randomly (lottery) selected during the period of April to June 2025. Then, households were randomly (using the lottery method) recruited within each district according to its population. One adult was randomly selected from each household; if there was no adult in the selected household, or if the available adults refused to participate, the next household was selected. 

#### 2.2.1. Inclusion and Exclusion Criteria

The study participants were adults aged 18 years or older residing in Atbara, whether they were displaced or not.

Exclusion criteria included individuals with a psychiatric diagnosis who were unable to provide informed consent, those who were sick, and pregnant or lactating women.

#### 2.2.2. Sample Size Calculation

OpenEpi software (version: 3.01, www.OpenEpi.com, updated 6 April 2013) was used to calculate the sample size [32]. A sample size of 373 adults was calculated assuming that 35.0% of adults would have depressive symptoms. This assumption was based on previous findings from Sudan that showed that half of the participants had depressive symptoms [33]. Then we assumed that 65.0% of adults with depressive symptoms would be internally displaced, and 50.0% of those without depressive symptoms would be internally displaced [34]. The calculated sample size (373 adults) would have 80% power and a precision of 0.05. 

### 2.3. Data Collection

After adults signed informed consent forms, data were collected by trained medical officers (medical college graduates) through face-to-face interviews using a structured questionnaire. The questionnaire included data on sociodemographic factors, such as age, gender, marital status, education status, employment status, smoking status, alcohol consumption, and history of hypertension and/or diabetes mellitus. Each adult’s weight and height were measured using a standard procedure, and their BMI was calculated as their weight in kilograms divided by the square of their height in meters. 

### 2.4. Outcome Measurements

The Arabic version of “Patient Health Questionnaire-9 (PHQ-9)” was used in several settings, including Sudan, with adequate validity, and was used in this study to assess depressive symptoms in adults [35,36,37]. It is a nine-item, widely used depressive-symptom-screening tool [38]. The instrument (PHQ-9) uses a four-point Likert-type scale, in which 0 stands for “Not at all” and 3 stands for “Almost every day.” Thus, the total scores range from 0 to 27, and the higher the PHQ-9 score, the higher the level of depressive symptoms [38]. Although a PHQ-9 cut-off value of ≥10 was used in some settings, in the current study, adults with PHQ-9 scores ≥ 8 were considered to have depressive symptoms, as reported in previous studies with acceptable accuracy/sensitivity and specificity [39,40]. The PHQ-9 questionnaire is a screening tool and not intended to make or confirm a diagnosis, which is the responsibility of the professional conducting the comprehensive clinical evaluation.

### 2.5. Statistical Analysis

The data were analyzed using IBM Statistical Product and Service Solutions (SPSS) for Windows (version 25.0; SPSS Inc., New York, NY, USA). After checking the continuous data (age, BMI, and depression score) for normality using the Kolmogorov–Smirnov test, they were found to be non-normally distributed and reported as medians (interquartile ranges, IQRs). Proportions were compared between adults with and without depressive symptoms using a chi-square test. Univariate binary analysis was used with depressive symptoms as the dependent variable. The independent variables were sociodemographics (age, gender, education level, marital status, occupation status, smoking habit, alcohol consumption, and internal displacement), BMI, diabetes mellitus, and hypertension. Multicollinearity was assessed (Variance Inflation Factor > 4) and not detected. These covariates/confounders were selected based on the literature and included in the final model (multivariate binary analysis) for adjustment when their *p*-values were <0.2 [41]. Missing data were handled with multiple imputation. Adjusted odds ratios (AORs) and 95% confidence intervals (CIs) were also calculated. A two-sided *p*-value of <0.05 was considered statistically significant. The Hosmer–Lemeshow test was used to assess the model’s goodness.

## 3. Results

### 3.1. General Characteristics of the Study Participants 

Three hundred and seventy-three adults were enrolled in this study; 104 (27.9%) were males. Their median (IQR) age and BMI were 42.0 (26.0–56.0) years and 25.8 (21.6–30.8) kg/m^2^, respectively. Of the enrolled 373 adults, 205 (55.0%) were married, and 239 (64.1%) had a secondary school education or above. About two-fifths (153; 41.0%) were employed. Of the total adults, 46 (12.3%) were cigarette smokers, and nine (2.4%) were alcohol consumers. One hundred and twenty-two (32.7%) of the adults were hypertensive, and 69 (18.5%) had diabetes mellitus. Of the enrolled 373 adults, 139 (37.3%) were internally displaced. The median (IQR) score for depression was 3 (1–7) (Table 1).

### 3.2. Prevalence and Factors Associated with Depressive Symptoms

Of the 373 adults, 81 (21.7%, 95.0% CI = 17.5–25.9%) had depressive symptoms (PHQ-9 scores ≥ 8) (Table 1). The prevalence of depressive symptoms was significantly higher among internally displaced adults; 39 (28.1%) of the 139 displaced adults had depressive symptoms, and 42 (17.9%) of the non-displaced adults had depressive symptoms, *p* = 0.027. 

Unadjusted (univariate) binary analysis showed that age (UOR = 0.98, 95.0% CI = 0.96–0.99), BMI (UOR = 0.95, 95.0% CI = 0.91–0.99), marital status (UOR = 1.95, 95.0% CI = 1.18–3.21), and internal displacement (UOR = 1.78, 95.0% CI = 1.08–1.93) were associated with depressive symptoms. In contrast, sex, education level, occupation, diabetes mellitus, hypertension, cigarette smoking, and alcohol consumption were not associated with depressive symptoms (Table 2).

Factors with *p* < 0.2 were included in the adjusted (multivariate) binary analysis. Being internally displaced was the only factor associated with depressive symptoms (AOR = 1.82, 95% CI 1.07–3.08); age, BMI, sex, education, and marital status were not associated with depressive symptoms (they were not confounding factors) (Table 3). The Hosmer–Lemeshow test indicated good model fit (*p* = 0.627).

## 4. Discussion

The main findings of the current study were that 21.7% of the adults had depressive symptoms, which was significantly higher (28.1%) among internally displaced adults. This is consistent with a previous study in Kasai, Democratic Republic of the Congo, where data were collected from 4069 heads of household (displaced and non-displaced) and showed that 18.3% of the heads of household had symptoms of depression, which was significantly higher among internally displaced (22%) than among non-displaced respondents (17%) [22]. The prevalence of depressive symptoms in this study was slightly higher than the prevalence (17.5%) reported in a community-based study in Ethiopia, which used ≥5 in the PHQ-9 as a cut-off point [6]. However, this prevalence of depressive symptoms among internally displaced adults was lower than the high prevalence reported in several previous studies [10,19,21,23,33,42]. For example, using an online questionnaire in Sudan, it has been found that over half the respondents had depressive symptoms, which was associated with female sex, financial loss, job loss, and interruption of education [33]. Another study in Sudan, in which the data were collected through online surveys and face-to-face interviews using the Beck Depression Inventory (BDI), showed that 53.5% of the participants had depression [10]. In a meta-analysis that included 132 and 195,741 participants, the pooled prevalence of depressive symptoms was 35.8%, and odds of depression were increased with internal displacement compared with non-displaced controls [23]. Following forced displacement due to armed conflict in Congo, it has been observed that 25.2% of the internally displaced adults in the camp had depression [42]. Of 750 participants, 63.8% of armed conflict survivors in Mozambique had depression [19]. Of 462 adults at the mean age of 41 years in Ukraine, 13.9% had depression before the war (2022), and this increased to 37.0% after being displaced after the war [21]. 

In this study, internal displacement persists as a primary, independent predictor of depressive symptoms. Thus, internal displacement, with its profound, sustained disruptions to a person’s previous life, overrides other sociodemographic factors. Displacement can lead to loss of financial security, social networks, housing, and autonomy [16]. Moreover, displacement can destroy immediate neighborhood networks and the community bonds that act as psychological buffers. Likewise, living in crowded, unfamiliar, or unsafe environments can continuously trigger the brain’s threat-detection mechanism, and hence can lead to depression [43]. Traumatic exposure combined with the loss of personal resources can disable a person’s ability to regulate their emotions and can increase the susceptibility to depressive symptoms [44].

This discrepancy may be attributed to differences between the studied populations, as the present study was conducted in the community of Atbara itself rather than among a displaced population in a camp. As mentioned above, although there were four camps for internally displaced people, we conducted this study in Atbara itself to provide context and a comparison. The participants were not displaced persons in refugee camps where precarious conditions, such as lack of water, food, and inadequate hygiene and sanitation, as well as the risk of communicable diseases, can play a greater role. All these factors affect the refugees’ health. The use of different diagnostic criteria or tools may also account for the variations observed when compared to other studies. The solution to treating depression resulting from war and displacement is not only medical; in these cases, it is not medication. The solution is more political, economic, and social, involving the peaceful resolution of conflicts with minimal impact on the population.

In the current study, age, sex, education, occupation, marital status, smoking, alcohol, and BMI were not associated with depression. In contrast, several previous studies have shown that age [6,9], being female [1,2,6,9,10], unmarried status [6,9], high body mass index (BMI) [11,12,13], having a chronic disease such as diabetes mellitus [14] or hypertension [2,3], having a low education level [8,10], unemployment [9,10], smoking habit [6], and alcohol consumption [2,15] have been reported to be associated with depressive symptoms in adults in different populations. Differences in socioeconomic status could explain the discrepancy between our results and other findings. 

### Limitations

The study was conducted in a single city in Sudan, and its results may not generalize to other parts of the country. Because this is a cross-sectional study, the findings should be described as associations rather than causal effects. Other mental illnesses such as anxiety and post-traumatic stress disorder were not assessed. The pre-displacement data on depressive symptoms were not available, and some patients could have been unaware of their mental illness. The duration of displacement, exposure to traumatic events, loss of relatives, financial hardship, housing conditions, social support, and access to mental health services were not assessed. Dietary factors, including an altered diet and microbiota, are other factors that may contribute to mood disorders [45]. This combination (diet and microbiota) could be common among refugee and displaced populations, which were not investigated [46]. 

## 5. Conclusions

One out of five adults in this region of Sudan had depressive symptoms, which was associated with internal displacement. More effort and further research are needed to explore possible interventions to improve mental health and livelihoods, especially among internally displaced people.

## Figures and Tables

**Table 1:** Characteristics of the enrolled participants from northern Sudan (n = 373), 2025.

Variable		Median	Interquartile Range
Age, years		42.0	26.0–56.0
Body mass index, kg/m^2^		25.8	21.6–30.8
Depression score (range 0–25)		3.0	1.0–7.0
		Frequency	Percentage
Sex	Male	104	27.9
	Female	269	72.1
Marital status	Married	205	55.0
	Unmarried	168	45.0
Education status	≥Secondary	239	64.1
	<Secondary	134	35.9
Occupation status	Employed	153	41.0
	Unemployed	220	59.0
Internally displaced	No	234	62.7
	Yes	139	37.3
Known diabetic	No	304	81.5
	Yes	69	18.5
Known hypertensive	No	251	67.3
	Yes	122	32.7
Cigarette smoking	No	327	87.7
	Yes	46	12.3
Alcohol consumption	No	364	97.6
	Yes	9	2.4
Depressive symptoms	No	292	78.3
	Yes	81	21.7

**Table 2:** Univariate analysis of factors associated with depressive symptoms among adults in North Sudan, 2025.

Variable		Depressive Symptoms		Odds Ratio (95% Confidence Interval)	*p*-Value
		Yes (Number = 81)	No (Number = 292)		
		Median (Interquartile Range)			
Age, years		33.0 (23.0–45.0)	40.0 (28.0–55.0)	0.98 (0.96–0.99)	0.008
Body mass index, kg/m^2^		24.2 (20.7–29.0)	26.2 (22.1–31.1)	0.95 (0.91–0.99)	0.025
		Frequency (%)	Frequency (%)		
Sex	Male	16 (19.8)	88 (30.1)	Reference	
	Female	65 (80.2)	204 (69.9)	1.75 (0.96–3.19)	0.067
Marital status	Married	34 (42.0)	171 (58.6)	Reference	
	Unmarried	47 (58.0)	121 (41.4)	1.95 (1.18–3.21)	0.009
Education status	≥Secondary	58 (71.6)	181 (62.0)	Reference	
	<Secondary	23 (28.4)	111 (38.0)	0.64 (0.37–1.10)	0.112
Occupation status	Employed	33 (40.7)	120 (41.1)	Reference	
	Unemployed	48 (59.3)	172 (58.9)	1.01 (0.61–1.67)	0.954
Internally displaced	No	42 (51.9)	192 (65.8)	Reference	
	Yes	39 (48.1)	100 (34.2)	1.78 (1.08–1.93)	0.023
Known diabetic	No	68 (84.5)	236 (80.8)	Reference	
	Yes	13 (16.0)	56 (19.2)	0.80 (0.41–1.56)	0.522
Known hypertensive	No	55 (67.9)	196 (67.1)	Reference	
	Yes	26 (32.1)	96 (32.9)	0.96 (0.57–1.73)	0.895
Cigarette smoking	No	73 (90.1)	254 (87.0)	Reference	
	Yes	8 (9.9)	38 (13.0)	0.73 (0.32–1.63)	0.449
Alcohol consumption	No	79 (97.5)	285 (97.6)	Reference	
	Yes	2 (2.5)	7 (2.4)	1.03 (0.21–5.06)	0.970

**Table 3:** Multivariate analysis of factors associated with depressive symptoms among adults in northern Sudan, 2025.

		Adjusted Odds Ratio (95.0% Confidence Interval)	*p*-Value
Age, years		0.99 (0.97–1.01)	0.262
Body mass index, kg/m^2^		0.97 (0.92–1.01)	0.180
Sex	Male	Reference	
	Female	1.86 (0.98–3.51)	0.055
Marital status	Married	Reference	
	Unmarried	1.62 (0.93–2.83)	0.087
Education status	≥Secondary	Reference	
	<Secondary	0.90 (0.48–1.65)	0.736
Internally displaced	No	Reference	
	Yes	1.82 (1.07–3.08)	0.026

## Data Availability

The data from the current study cannot be provided due to privacy and ethical reasons.

## Abbreviations

The following abbreviations are used in this manuscript:

Kg	kilogram
M	meter
CM	centimeter
BMI	body mass index
CI	confidence interval
SPSS	Statistical Package for the Social Sciences
IQR	interquartile range
WHO	World Health Organization
